# Post-Translational Modifications of G Protein–Coupled Receptors Revealed by Proteomics and Structural Biology

**DOI:** 10.3389/fchem.2022.843502

**Published:** 2022-03-10

**Authors:** Bingjie Zhang, Shanshan Li, Wenqing Shui

**Affiliations:** ^1^ iHuman Institute, ShanghaiTech University, Shanghai, China; ^2^ School of Life Science and Technology, ShanghaiTech University, Shanghai, China

**Keywords:** G protein couped receptors, mass spectrometry-based proteomics, Post-translational modification (PTM), phosphorylation, signaling regulation

## Abstract

G protein–coupled receptors (GPCRs) are a protein superfamily comprising >800 members that regulate numerous cellular and physiologic responses. GPCRs represent the largest class of therapeutic targets with implications in various diseases. Although advances in GPCR structural and pharmacological research have significantly improved our knowledge of GPCR signaling mechanisms, mapping diverse post-translational modifications (PTMs) of GPCR proteins and understanding their regulatory roles have received much less attention. Mass spectrometry-based proteomics has become the most popular technology for profiling protein PTMs in a systematic manner. Herein we provide an overview of PTM types, locations, crosstalk and dynamic regulation for different GPCRs that are characterized using proteomic and/or biochemical approaches. Our main focus is on glycosylation, phosphorylation, ubiquitination and palmitoylation that are known to modulate receptor folding, biosynthesis, trafficking, dimerization and signaling. Furthermore, we discuss the locations of specific PTM sites in the structure of a given GPCR and its signaling complex to highlight the importance of PTM regulation in the molecular basis of GPCRs, which may shed new light on structure-based drug discovery.

## Introduction

G protein-coupled receptors (GPCRs), which are seven-transmembrane proteins, constitute the largest family of cell surface receptors in mammalian cells (>800 in human). According to sequence homology, mammalian GPCRs are divided into five major subfamilies: Rhodopsin (Class A), Secretin/Adhesion (Class B), Glutamate (Class C), Frizzled (Class F) and Taste 2 (Class T) (Lagerstrom and Schioth, 2008). GPCR activation results in coupling to G proteins at the plasma membrane and signaling from endosomes after receptor internalization (Thomsen et al., 2016). Dysregulation of GPCR signaling contributes to various human diseases such as obesity, diabetes, depression, Alzheimer’s disease and multiple types of cancer (Dorsam and Gutkind, 2007; Hauser et al., 2017; Huang et al., 2017; Zhou and Wild, 2019). Serving as the most successful drug target family, GPCRs currently account for targets of over 34% of FDA approved therapeutics (Kooistra et al., 2021).

Post-translational modifications (PTMs) mediate the abundance and/or activity of vast proteins and thus play a critical role in modulating signal transduction. Through covalently attaching a chemical or protein moiety to specific sites, PTMs increase the functional diversity of proteins and fine-tune signaling cascades (Krishna and Wold, 1993). These modifications including phosphorylation, glycosylation, ubiquitination, palmitoylation, methylation, acetylation and lipidation affect almost all aspects of normal cell biology and pathogenesis. For GPCRs, the most extensively characterized PTMs are glycosylation, phosphorylation, ubiquitination and palmitoylation which control the spatial and temporal dynamics of receptor signaling and physiologic responses (Duarte and Devi, 2020; Patwardhan et al., 2021). Specific PTMs are known to regulate receptor folding, maturation, trafficking, dimerization, and signaling activity (Patwardhan et al., 2021). Disorders of GPCR PTMs, which cause deficient or overabundant signaling responses, are linked to a variety of diseases, such as neurodegenerative disorders (Fang et al., 2020), immune dysfunction (Farzan et al., 1999) and cancer (Chen et al., 2017; Xiao et al., 2017).

**FIGURE 1:**
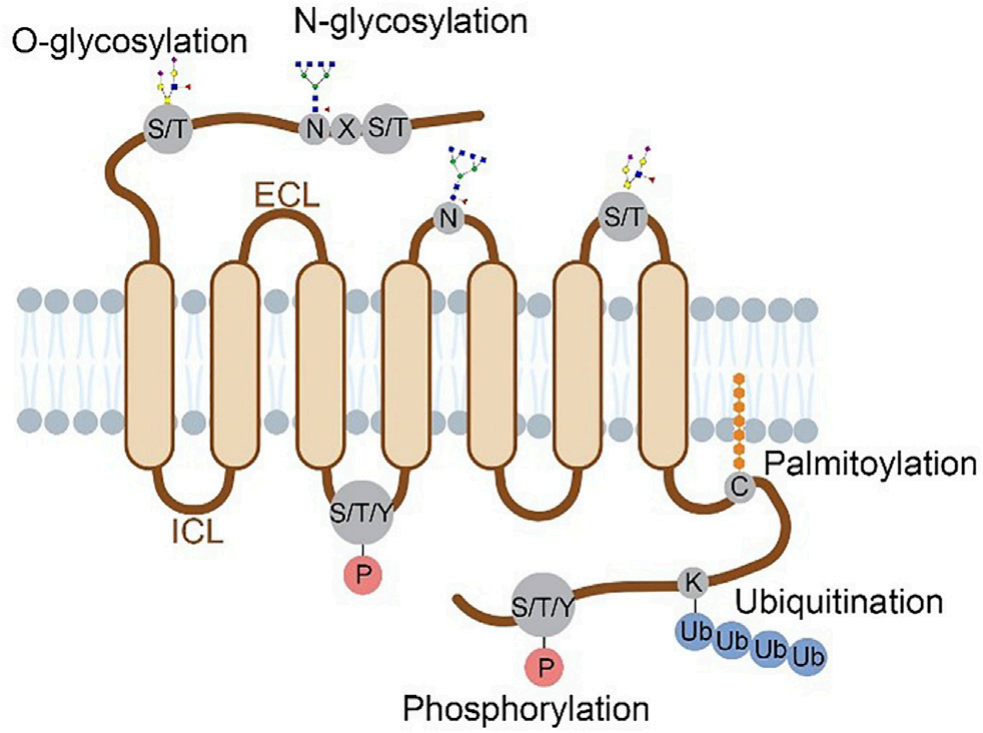
Structural localization of GPCR post-translational modifications overviewed in this review. Four major types of PTMs are distributed on the N-terminus, ECLs, ICLs and C-terminus of a GPCR protein. Glycosylation occurs on the N-terminal and ECL domains, with N-glycosylation at N of the sequence motif N-X-S/T (X≠P) and O-glycosylation at S/T residues. Phosphorylation occurs at S, T or Y residues on the C-terminal and ICL domains. Ubiquitination occurs at K residues and palmitoylation at C residues, both on the C-terminus.

The characterized GPCR PTMs occur at different domains of the receptor including N-terminus, extracellular loops (ECLs), intracellular loops (ICLs) and C-terminus. Compared to the seven transmembrane domains, these regions are much more accessible to PTM enzymes. Figure 1 summarizes the locations of multiple PTM types in the extracellular or cytoplasmic regions of a GPCR protein. Historically, these PTM types were discovered using metabolic labeling with radioactive probes or enzymatic methods, and PTM sites were deduced by site-directed mutagenesis (Prihandoko et al., 2015; Lu and Fang, 2020). More recently, prominent advancements in mass spectrometry (Nguyen et al.)-based proteomics allow for systematic analysis of protein PTM sites and abundances in cultured cells and tissues (Olsen and Mann, 2013; Hansen et al., 2021; Kitata et al., 2021), which facilitates PTM profiling in various GPCR proteins. In this review, we provide an overview of PTM types, locations, crosstalk and dynamic regulation for different GPCR proteins that are characterized mainly with proteomic approaches. In addition, specific PTMs revealed by structural biology are highlighted to understand the importance of PTMs in regulating the molecular function of GPCRs.

## GPCR Glycosylation

Glycosylation mainly takes place in the endoplasmic reticulum (ER) and Golgi apparatus and serves as a tag to direct the receptor to the plasma membrane (Schjoldager et al., 2020). N- and O-linked glycosylation are prevalently present at the N-terminal or ECL domains of GPCRs, and both modulate receptor maturation, trafficking, ligand binding and cell signaling (Chen et al., 2010; Lackman et al., 2018; Wang et al., 2020). N-glycosylation which links a sugar molecular to the nitrogen of Asn (N) residue in the consensus motif N-X-S/T (X≠P) is the major form of glycosylation found in GPCRs. Traditionally, enzymatic cleavage with PNGase F or Endo H to remove glycans along with site-directed mutagenesis is widely employed to detect N-glycosylation of specific receptors. For instance, three sites of GLP-1R (N63, N82 and N115) and four sites of mGluR7 (N98, N458, N486, N572), all at the N-terminus, were found to be glycosylated using this approach (Chen et al., 2010; Irwin et al., 2012; Park et al., 2020). Combined mutation of 2-3 glycosites caused significant reduction of GLP-1R cell surface expression, indicating that the cooperative function of multiple glycosites (Chen et al., 2010; Irwin et al., 2012).

Although biochemical methods have been effective for mapping glycosylation sites of specific GPCRs in an overexpression system, comprehensive profiling of glycosites and glycan compositions of various receptors *in vivo* remain very difficult because of the structural complexity and varying abundance of glycans. Fortunately, development of new methods for the enrichment and MS analysis of glycopeptides has largely promoted systematic profiling of various glycoproteins including GPCRs. Zielinska *et al.* developed a ‘‘filter aided sample preparation’’ (FASP) method to enrich glycopeptides bound to lectins on top of a filter (Zielinska et al., 2010). After removal of the N-glycans, peptides were analyzed by high-resolution MS to determine their N-glycosites. This work identifeid 6367 N-glycosites on 2352 proteins in four mouse tissues and plasma, among which many novel glycosites were detected for tissue-specific proteins, such as neurotransmitter receptors and contactins in the brain. Liu *et al.* employed zwitterioic hydrophilic interaction liquid chromatography (ZIC-HILIC) for separating and enriching glycopeptides (Liu et al., 2017). They analyzed intact N-glycopeptides at the proteome scale using a stepped collision energy-based MS method. The MS data were processing with a dedicated search engine pGlyco 2.0 to decode the N-glycosites and N-glycan composition simultaneously. This study generated a large-scale glycoproteome dataset consisting of 10,009 site-specific N-glycans on 1988 glycosites from 955 glycoproteins in five mouse tissues. Of note, the two previous glycoproteomic datasets included a small fraction of glycosylated GPCRs identified in different mouse tissues (180 N-glycosites mapped to 84 GPCRs by Zielinska *et al.*, and 51 N-glycosites mapped to 26 GPCRs by Liu *et al.*). A similar approach of intact glycopeptide analysis was adopted by Fang *et a*l. to map the brain N-glycoproteomic landscape in an AD mouse model (Fang et al., 2020). Interestingly, among the hundreds of up- or down-regulated N-glycopeptides from the AD mouse brain relative to the control, we noticed that 25 N-glycopeptides mapped to 8 GPCRs such as S1P1, Gpr158, mGluR3 showed dysregulated glycosylation occupancy on specific sites.

Apart from the direct glycopeptide enrichment, capturing cell-surface proteins prior to glycopeptide enrichment is another approach to improve the sensitivity of profiling low-abundance N-glycosylation. Through covalently labeling extracellular glycan moieties in live cells, Danzer *et al.* identified N-glycosites of 28 GPCRs in mouse pancreatic β-cells or human islets (Danzer et al., 2012). These glycosylated GPCRs span orphan receptors (e.g., GPR116, GPR158), class A (e.g., ADRA2A, GALR1), class B (e.g., GLP-1R, CRHR1), and class C (e.g., CASR, GABBR2) receptors. For GLP-1R which represents an important therapeutic target of type II diabetes and obesity, glycosylation at both N63 and N115 were detected in this study. Moreover, glycopeptides released from these two sites were reduced by 9-fold in response to glucose and GLP-1 stimulation as measured by quantitative MS analysis, implying the involvement of N-glycosylation of GLP-1R in insulin secretion and blood glucose control.

In regard to mucin-type O-linked glycosylation, up to 20 different GalNAc transferases installs N-acetylgalactosamine to the hydroxyl group of S, T or Y residues in Golgi after protein folding. Different monosaccharides are then added successively to the growing oligosaccharide before the elongated glycans are capped with terminal sialic acids (Patwardhan et al., 2021). Lack of a consensus sequence and enzymatic tools, together with highly complex and heterogeneous glycan structures, makes it more difficult to determine the modification site and glycan composition of O-glycosylation than N-glycosylation (Wilkinson and Saldova, 2020). Although computational prediction implicates over 350 GPCRs could be O-glycosylated, most of them are not experimentally verified (Steentoft et al., 2013). To reduce the complexity of O-glycosylation in cells, Steentoft *et al.* developed a genetic engineering strategy to produce truncated and homogeneous O-glycans by blocking the elongation process. The simplified O-linked glycopeptides were then analyzed with an HCD/ETD hybrid MS method to determine the O-glycosylation sites on various peptides (Steentoft et al., 2011). Applying this strategy to O-glycoproteome profiling of 12 human cell lines generated an expanded map of almost 3,000 glycosites from over 600 O-glycoproteins (Steentoft et al., 2013). From this dataset, we found 35 O-glycosites mapped to 14 GPCRs. For instance, 5 O-glycosites were identified in Frizzled-2 receptor (FZD2) and 3 O-glycosites in adhesion receptor GPR64, all within the N-terminal domain. Recently a new chemoenzymatic method named EXoO was introduced for the selective extraction of O-linked glycopeptides from protein digests. Yang *et al.* exploited an endo-protease OpeRATOR to specifically release O-glycopeptides from proteins conjugated to a solid support before their glycosites were assigned by high-resolution MS/MS analysis (Yang et al., 2018). EXoO was benchmarked with human kidney tissue, T cells, and serum to map a total of 3,055 O-glycosites from 1,060 glycoproteins, which included 23 GPCRs with 39 O-glycosites assigned.

A profound breakthrough in the glycoproteomics field is the recent development of a panel of bioinformatics tools such as Byonic (Bern et al., 2012), GPQuest (Toghi Eshghi et al., 2015), pGlyco3 (Zeng et al., 2021) and StrucGP (Shen et al., 2021) for efficient interpretation of tandem MS data from N-linked or O-linked glycopeptides. These search engines enable accurate identification of the composition and localization of glycans on glycopeptides as well as elucidation of site-specific glycan structures on a proteome-wide scale. Major characteristics of these software tools are summarized in Table 1. We envision such bioinformatic advances would facilitate the structural and functional study of GPCR glycosylation.

**TABLE 1:** Major characteristics of different glycoproteomic search engines.

	Byonic	GPQuest	pGlyco	StrucGP
Open source	no	yes	yes	yes
Graphical interface	yes	yes	yes	yes
Intact N-glycopeptide identification^a^	yes	yes	yes	yes
Intact O-glycopeptide identification^a^	yes	yes	yes	no
Glycan structure^b^	no	no	no	yes
Glycan database^c^	yes	yes	yes	no

a Able to determine both N-/O-glycosites and glycan composition.

b Able to detemine the detailed glycan structures on specific sites.

c Dependent on a glycan database or not when searching MS data.

### GPCR Phosphorylation

Phosphorylation is a major regulator of GPCR transduction signaling dynamics in mammalian cells. GPCR phosphorylation mainly mediated by two classes of serine/threonine kinases, namely GPCR kinases and second message kinases (such as protein kinase A and protein kinase C) (Lefkowitz, 1998). A large number of GPCR phosphorylation sites have been reported, mostly using mass spectrometry, phosphor-specific immunoblotting and metabolic labeling approaches (Prihandoko et al., 2015).

The traditional method for detecting GPCR phosphorylation in early studies is through metabolic labeling of cultured cells with the radioactive phosphate (usually ^32^P orthophosphate) (Fredericks et al., 1996; Prihandoko et al., 2015). Metabolic labeling employed together with receptor–specific immunoprecipitation provides a global assessment of GPCR phosphorylation. However, this approach does not allow precise mapping of phosphorylated residues, which requires mass spectrometry or phosphosite-specific antibodies.

Compared to immunoblotting with site-specific antibodies, MS-based proteomic profiling enables more comprehensive site determination and accurate quantification of protein phosphophorylation *in vitro* and *in vivo* (Lawrence et al., 2016; Liu et al., 2018). This powerful technology has been applied to phosphorylation mapping and signaling investigation of numerous GPCRs. In the case of β_2_-adrenergic receptor (β_2_AR) expressed in HEK293 cells, by performing MS analysis of phosphosite-specific regulation, the authors reported the induction of phosphorylation at 13 sites located at ICL3 or C-terminus by an unbiased agonist (Nobles et al., 2011). Distinct phosphorylation only occurred at S355 and S356 in response to the stimulation with a β-arrestin-biased agonist carvedilol, which was discovered by MS analysis and validated with site-specific antibodies (Nobles et al., 2011). Application of the same strategy has led to decoding 14 phosphorylation sites on M1 muscarinic acetylcholine receptor (M1 mAChR) upon the simulation of acetylcholine (Butcher et al., 2016). Although S228 in ICL3 of M1 mAChR displayed an extremely low level of constitutive phosphorylation, its modification level was dramatically up-regulated under the stimulation of an orthosteric agonist. A positive allosteric modulator was able to further enhance acetylcholine-induced phosphorylation at S228. The MS-based proteomic approach has also been applied to mapping three endogenous phosphosites of MOP in the mouse brain (Mouledous et al., 2015). Only the phosphorylation of T370 and S375 was enhanced by agonist administration *in vivo*.

**FIGURE 2:**
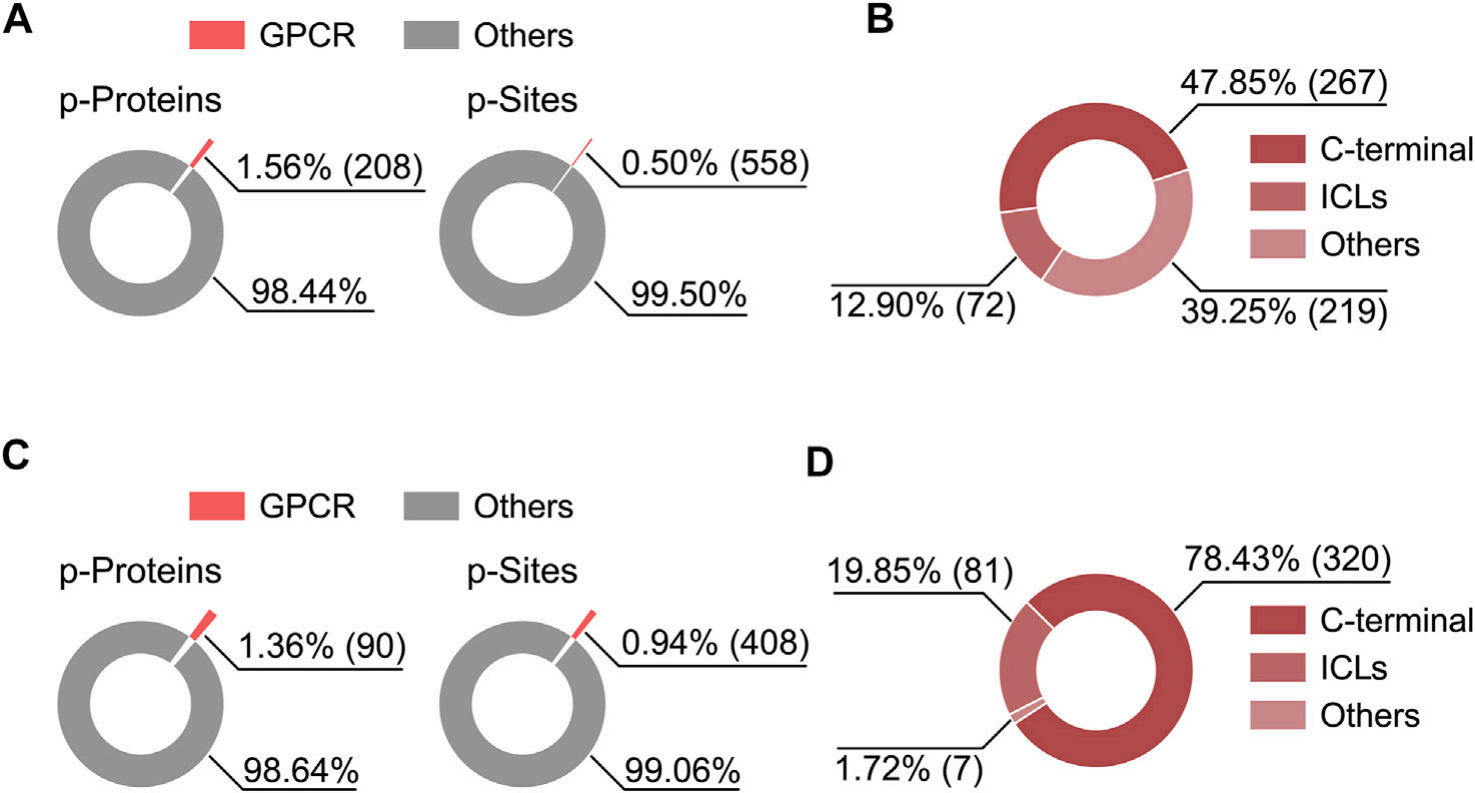
Percentage of phosphorylated GPCRs and their phosphosites in the total identifications from two published phophosproteomics datasets **(A,C)**, and the distribution of identified phosphosites on GPCR topological domains **(B,D)**. **(A,B)** Results based on a human phosphoproteome dataset (Lawrence et al., 2016). **(C,D)** Results based on a mouse brain phosphoproteomics dataset (Liu et al., 2018). Criteria for phosphoprotein and phosphosite identification are PSM FDR <0.01, phosphopeptide FDR <0.05, and Ascore >13 at the phosphosite level for **(A,B)**, and PSM FDR <0.01, protein FDR <0.01, and phosphosite localization probability >0.75 for **(C,D)**.

MS-based phosphoproteomics has become the method of choice for the genome-wide study of protein phosphorylation and dynamic cell signaling (Humphrey et al., 2015). Recently, Liu *et al.* employed high-throughput phosphoproteomics to study *in vivo* signaling of kappa opioid receptor (KOR) induced by structurally diverse agonists in five mouse brain regions (Liu et al., 2018). By analyzing this proteomics dataset, we noticed the phosphorylation level at S317 of cannabinoid receptor 1 (CB1) was downregulated upon KOR’s aversive agonist administration, suggesting a signaling crosstalk might be present between CB1 and KOR through dynamic regulation of phosphorylation. We also analyzed the percentage of phosphorylated GPCRs and their phosphosites in total identifications as well as topological localization of identified phosphosites in GPCRs included in this dataset and in a most comprehensive human phosphoproteome database (Christensen et al., 2010; Lawrence et al., 2016) (Figure 2). Among the 6766 phosphoproteins reported in the KOR signaling study, a very small fraction (1.36%) was phosphorylated GPCRs (Figure 2C), highlighting the challenge of detecting endogenous GPCR phosphorylation in tissues.

Given that the C-terminus of GPCRs often contains multiple serine, threonine and tyrosine residues, the hydrophilic phosphopeptides released from this region are easily overlooked in conventional proteomic workflows. To address this issue, a TMT chemical labeling method was developed to increase the phosphopeptide hydrophobicity so as to render quantitative measurement of the phosphorylated C-terminus of a selected GPCR (Tsai et al., 2019). Using CXCR3 as an example, both its unphosphorylated and single-site phosphorylated form at the C-terminus were detected and quantified under agonist stimulation. This method is anticipated to expand the coverage of GPCR phosphoproteome profiling.

### GPCR Ubiquitination

GPCR ubiquitination is an enzymatic process that mediates the covalent conjugation of ubiquitin to a targeted protein. This process is critical for regulating biosynthesis, endocytosis, lysosomal sorting degradation and cellular signaling of GPCRs (Kennedy and Marchese, 2015; Patwardhan et al., 2021). In general, GPCRs are modified at one or multiple intracellular lysine residues with either monoubiquitin or polyubiquitin chains in an agonist-dependent or -independent manner (Imai et al., 2001; Marchese and Benovic, 2001). Currently, the major strategy adopted for profiling GPCR ubiquitination is target protein immunoprecipitation followed by immunoblotting or MS-based proteomic analysis. Immunoblotting coupled with mutagenesis of targeted lysine residues was performed to infer the ubiquitin-conjugation sites on mGluR7 (Lee et al., 2019). Two lysine residues at the ICL2 and eight lysine residues at the C terminus of mGluR7 were found to be ubiquitinated, which was primarily mediated by Nedde E3 ligase with agonist treatment. Complementary to immunoblotting and mutagenesis, proteomic analysis allows for direct and systematic identification of all putative ubiquitination sites in a specific GPCR. To study the regulation of β_2_AR ubiquitination, Xiao *et al.* performed LC-MS/MS analysis of tryptic digests of the purified receptor with or without agonist stimulation (Xiao and Shenoy, 2011). Lysines at the ICL3 (K263 and K270) and the C-terminus (K348, K372 and K375) of β_2_AR showed agonist-induced ubiquitination, which played a key role in the long-term receptor desensitization through lysosomal degradation. Using the same approach, Zhang *et al.* reported characteristic ubiquitin modifications of specific residues at the ICL3 (K388) and the C-terminus (K484) of parathyroid hormone receptor (PTHR) upon PTH (1–34) stimulation (Zhang et al., 2018). These two ubiquitination sites were then confirmed by site-directed mutagenesis and shown to modulate PTHR trafficking, signaling and function in HEK293 cells.

### GPCR Palmitoylation

GPCR palmitoylation involves covalent attachment of palmitate (saturated 16-carbon fatty acid) to one or more cysteine residues of the receptor via a thioester bond (S-palmitate). This modification usually occurs basally at the C-terminus of GPCRs during their biosynthesis, and in some cases, can be induced by agonist stimulation (Qanbar and Bouvier, 2003). GPCR palmitoylation plays an important role in receptor trafficking, localization to cell surface, dimerization and signaling (Qanbar and Bouvier, 2003; Chini and Parenti, 2009). The analysis of GPCR palmitoylation remains challenging due to the hydrophobicity and instability of S-palmitate modified peptides. Recently, bioorthogonal labeling or click chemistry have been increasingly used for GPCR palmitoylation profiling in receptor-overexpressing cell lines (Zuckerman et al., 2011; Adachi et al., 2016). One of these methods is to use alkyne-modified palmitic acid reporters to label proteins in live cells and then apply azide biotin for isolation or immunoblotting of palmitoylated proteins from cell lysates (Ebersole et al., 2014; Kallemeijn et al., 2021). Notably, metabolic labeling or chemical labeling based on acyl-biotin replacement or acyl-polyethylene glycol (PEG) exchange have been developed to achieve sensitive analysis of protein S-palmitoylation sites and abundances on a proteome-wide scale (Yang et al., 2010; Percher et al., 2016). Similar to phophoproteomics, palmitoylation sites in a number of GPCRs such as FZD5, GPRC5A and mGluR7 were determined in a proteome-wide analysis of human palmitoylated proteins (Yang et al., 2010).

### GPCR PTM Crosstalk

Typically, GPCRs are modified at multiple sites by various PTMs to regulate their structure, stability, activity and function. However, these PTMs do not exist in isolation and they can either positively or negatively influence each other. This combinatorial effect of different PTMs at the same or multiple residues is termed PTM crosstalk. PTM crosstalk offers unique mechanisms for GPCR functional regulation. The crosstalk between GPCR phosphorylation and ubiquitination has been extensively studied. For instance, mutation of phosphorylated residues S324 and S325 of CXCR4 inhibited agonist-induced ubiquitination of nearby lysine residues and eventually affected receptor degradation. This was attributed to the impaired recruitment of E3 ubiquitin ligase AIP4 to the cell membrane by the phosphor-deficient mutants (Bhandari et al., 2009). In another work, mutation of phosphorylated residues T387 and T392 of PTHR suppressed β-arrestin recruitment after agonist activation and inhibited subsequent PHTR ubiquitination (Zhang et al., 2018). In fact, it is proposed that most of GPCR agonist-induced ubiquitination requires phosphorylation for direct recruitment of the E3 ligase or adaptor proteins that mediate the interaction or activity of the ubiquitination machinery. However, a comprehensive proteomic map and mechanistic details of interdependent GPCR phosphorylation and ubiquitination remain unavailable.

### PTMs Observed in High-Resolution GPCR Structures

**FIGURE 3:**
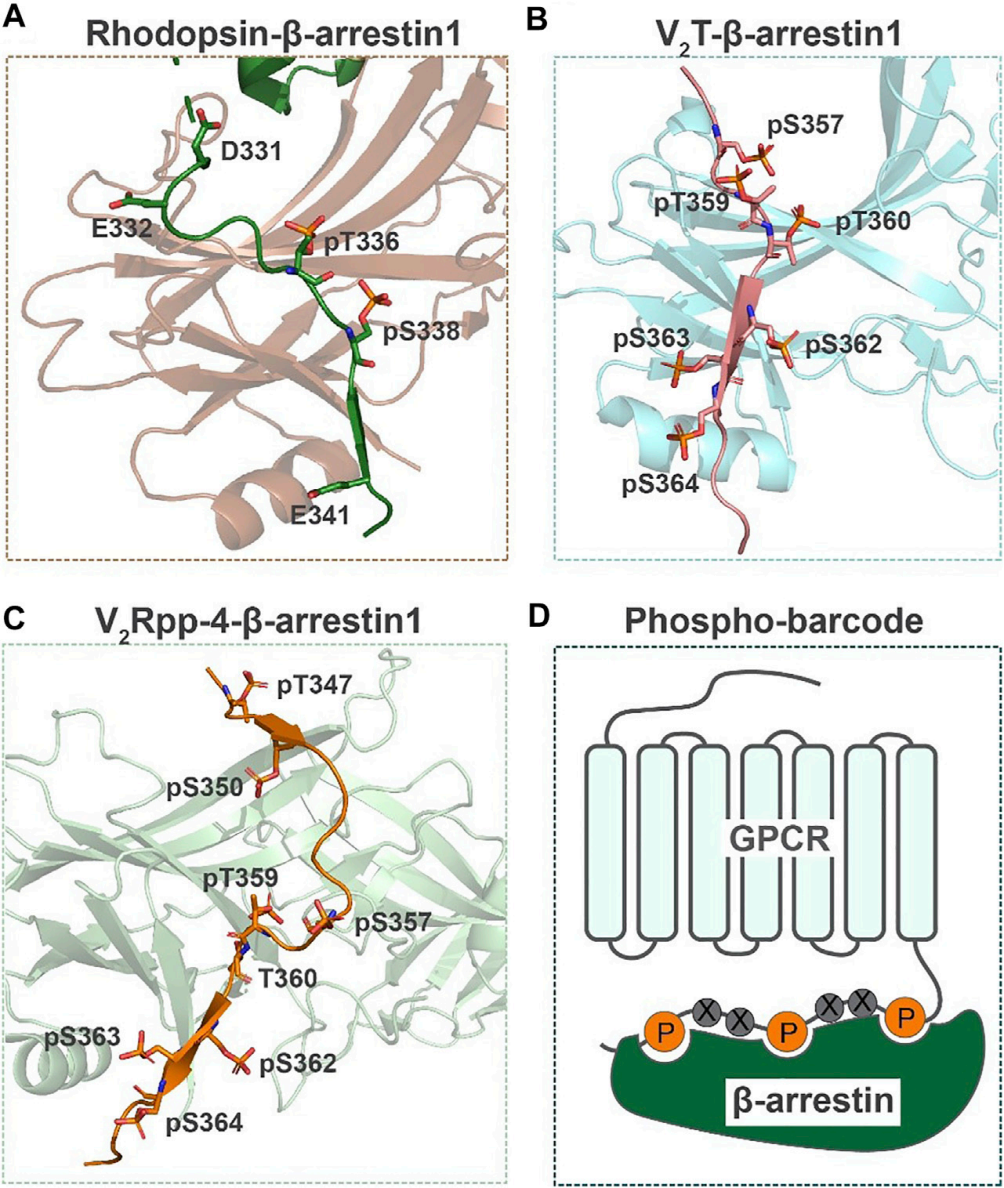
GPCR phosphorylation sites present in crystal or cryo-EM structures. **(A)** The phosphorylated rhodopsin C terminus (green) interacting with β-arrestin1 (brown) in the crystal structure (Zhou et al., 2017). **(B)** Phosphorylated residues on V_2_T (pink) interacting with β-arrestin1 (light blue) in the cryo-EM structure (Nguyen et al., 2019). **(C)** Crystal structure of β-arrestin1 (light green) in complex with a V_2_R phosphopeptide (orange) (He et al., 2021). **(D)** A model of the GPCR phosphorylation code in a pattern of *P*x(x)*P*xx*P* for β-arrestin recruitment (Zhou et al., 2017).

Over the last decade, technology breakthroughs in structural biology of membrane proteins have resulted in the determination of over 600 structures of GPCRs in complexes with various ligands and signaling partners. However, PTM moieties are rarely present in these high-resolution structures possibly due to the fact that the majority of PTMs are located in highly flexible regions such as the N- and C-terminus. Nevertheless, in the X-ray free electron laser (XFEL) crystal structure of a rhodopsin-arrestin complex, phosphorylation of two residues T336 and S338 of rhodopsin were observed (Zhou et al., 2017) (Figure 3A). The two phosphosites, together with E341, formed an electrostatic interaction network with three positively charged pockets in β-arrestin1 to stabilize the entire complex. Moreover, the authors proposed a phosphorylation code in the receptor C-tail as a common mechanism of mediating arrestin recruitment (Zhou et al., 2017) (Figure 3D). More recently, in the cryo-EM structure of β_2_V_2_R-G protein-β-arrestin1 megaplex, six GRK2-phosphorylated residues were observed in the subcomplex of β-arrestin1 and vasopressin receptor-2 C-tail (V_2_T) (Nguyen et al., 2019) (Figure 3B). The presence of multiple phosphosites on the V_2_T presumably enhanced the affinity of β_2_V_2_R with β-arrestin1 to form the megaplex. The majority of these phosphosites on V_2_T made electrostatic interactions with lysine or arginine residues at the N-terminus of β-arrestin1. Through MS analysis of the phosphorylation code on the V_2_T, this study revealed that four residues in this domain were basally phosphorylated, whereas the other four residues were phosphorylated in response to agonist stimulation. These results indicated that a specific C-terminal phosphorylation pattern of GPCR is required for recruiting and stabilizing the signaling transducer β-arrestin1.

To further elucidate the mechanism of regulating arrestin interaction and function by GPCR phosphorylation codes, He *et al.* determined the structures of arrestin2 in complex with four different phosphopeptides derived from the V_2_T (He et al., 2021) (one representative structure shown in Figure 3C). These crystal structures in line with NMR analysis and functional characterization suggested that different phosphorylation patterns of a GPCR could not only determine the strength of the phosphor-arrestin interaction, but also induce distinct conformational changes at remote positions of arrestin to ultimately modulate its selective functions.

In addition to phosphorylation, palmitoylation at cysteine residues observed in both structures of rhodopsin and adrenoceptor β_2_AR (Salom et al., 2006; Cherezov et al., 2007). For instance, in the β_2_AR crystal structure, C341 on the receptor H8 helix was modified with a palmitic acid, which makes hydrophobic interaction with cholesterol to possibly regulate receptor dimerization (Cherezov et al., 2007).

## Conclusion and Perspective

Diverse PTMs in GPCR proteins provide novel and expansive mechanisms for GPCR functional regulation as well as new opportunities for GPCR-targeted drug development (Patwardhan et al., 2021). With the advancement of MS-based proteomics technology, a number of PTM sites have been mapped to specific receptors stimulated with different ligands, which substantially enhanced our mechanistic understanding of receptor trafficking, activation, internalization and degradation. Most biochemical studies looked into one receptor at a time, identified PTM residues by MS analysis, and elucidated the function of specific modification sites in GPCRs overexpressed in cell lines. Furthermore, certain PTMs are observed in GPCR structures, providing a molecular basis for *in vitro* PTM regulation of receptor conformation and interaction with signal transducers.

However, GPCR PTM characterization at physiological conditions remains a long-standing challenge due to the low receptor expression and low stoichiometry of most PTMs in primary cells or tissues. Therefore, more sensitive and robust techniques are required for mapping PTM sites on endogenous GPCRs and profiling the spatial and temporal dynamics of GPCR PTMs during disease progression. Revealing the PTM landscape of various GPCRs at pathological conditions would foster our understanding of dysregulated mechanisms in diseases and discovery of new drug targets. It is noteworthy that a growing number of large-scale PTM proteomic studies have documented GPCR modification sites and regulation, but they are lack of functional linkage to specific receptors. More GPCR-oriented proteomic studies need to be designed to uncover new regulatory mechanisms and physiological functions for GPCR PTMs in a systematic manner.

## References

[B1] Adachi N. Hess D. T. Mclaughlin P. Stamler J. S. (2016). S-Palmitoylation of a Novel Site in the β2-Adrenergic Receptor Associated with a Novel Intracellular Itinerary. J. Biol. Chem. 291, 20232–20246. 10.1074/jbc.M116.725762 27481942 PMC5025705

[B2] Bern M. Kil Y. J. Becker C. (2012). Byonic: Advanced Peptide and Protein Identification Software. Curr. Protoc. Bioinformatics 13, 20. 10.1002/0471250953.bi1320s40 23255153 PMC3545648

[B3] Bhandari D. Robia S. L. Marchese A. (2009). The E3 Ubiquitin Ligase Atrophin Interacting Protein 4 Binds Directly to the Chemokine Receptor CXCR4 via a Novel WW Domain-Mediated Interaction. Mol. Biol. Cel 20, 1324–1339. 10.1091/mbc.E08-03-0308 PMC2649280 19116316

[B4] Butcher A. J. Bradley S. J. Prihandoko R. Brooke S. M. Mogg A. Bourgognon J.-M. (2016). An Antibody Biosensor Establishes the Activation of the M1 Muscarinic Acetylcholine Receptor during Learning and Memory. J. Biol. Chem. 291, 8862–8875. 10.1074/jbc.M115.681726 26826123 PMC4861454

[B5] Chen Q. Miller L. J. Dong M. (2010). Role ofN-Linked Glycosylation in Biosynthesis, Trafficking, and Function of the Human Glucagon-Like Peptide 1 Receptor. Am. J. Physiology-Endocrinology Metab. 299, E62–E68. 10.1152/ajpendo.00067.2010 PMC2904048 20407008

[B6] Chen S. Zhu B. Yin C. Liu W. Han C. Chen B. (2017). Palmitoylation-Dependent Activation of MC1R Prevents Melanomagenesis. Nature 549, 399–403. 10.1038/nature23887 28869973 PMC5902815

[B7] Cherezov V. Rosenbaum D. M. Hanson M. A. Rasmussen S. G. F. Thian F. S. Kobilka T. S. (2007). High-Resolution Crystal Structure of an Engineered Human β 2 -Adrenergic G Protein-Coupled Receptor. Science 318, 1258–1265. 10.1126/science.1150577 17962520 PMC2583103

[B8] Chini B. Parenti M. (2009). G-Protein-Coupled Receptors, Cholesterol and Palmitoylation: Facts about Fats. J. Mol. Endocrinol. 42, 371–379. 10.1677/JME-08-0114 19131499

[B9] Christensen G. L. Kelstrup C. D. Lyngsø C. Sarwar U. Bøgebo R. Sheikh S. P. (2010). Quantitative Phosphoproteomics Dissection of Seven-Transmembrane Receptor Signaling Using Full and Biased Agonists. Mol. Cell Proteomics 9, 1540–1553. 10.1074/mcp.M900550-MCP200 20363803 PMC2938087

[B10] Danzer C. Eckhardt K. Schmidt A. Fankhauser N. Ribrioux S. Wollscheid B. (2012). Comprehensive Description of the N-Glycoproteome of Mouse Pancreatic β-Cells and Human Islets. J. Proteome Res. 11, 1598–1608. 10.1021/pr2007895 22148984

[B11] Dorsam R. T. Gutkind J. S. (2007). G-Protein-Coupled Receptors and Cancer. Nat. Rev. Cancer 7, 79–94. 10.1038/nrc2069 17251915

[B12] Duarte M. L. Devi L. A. (2020). Post-Translational Modifications of Opioid Receptors. Trends Neurosciences 43, 417–432. 10.1016/j.tins.2020.03.011 PMC7323054 32459993

[B13] Ebersole B. Petko J. Levenson R. (2014). Bioorthogonal Click Chemistry to Assay Mu-Opioid Receptor Palmitoylation Using 15-Hexadecynoic Acid and Immunoprecipitation. Anal. Biochem. 451, 25–27. 10.1016/j.ab.2014.01.008 24463015 PMC3969759

[B14] Fang P. Xie J. Sang S. Zhang L. Liu M. Yang L. (2020). Multilayered N-Glycoproteome Profiling Reveals Highly Heterogeneous and Dysregulated Protein N-Glycosylation Related to Alzheimer's Disease. Anal. Chem. 92, 867–874. 10.1021/acs.analchem.9b03555 31751117

[B15] Farzan M. Mirzabekov T. Kolchinsky P. Wyatt R. Cayabyab M. Gerard N. P. (1999). Tyrosine Sulfation of the Amino Terminus of CCR5 Facilitates HIV-1 Entry. Cell 96, 667–676. 10.1016/s0092-8674(00)80577-2 10089882

[B16] Fredericks Z. L. Pitcher J. A. Lefkowitz R. J. (1996). Identification of the G Protein-Coupled Receptor Kinase Phosphorylation Sites in the Human β2-Adrenergic Receptor. J. Biol. Chem. 271, 13796–13803. 10.1074/jbc.271.23.13796 8662852

[B17] Hansen F. M. Tanzer M. C. Brüning F. Bludau I. Stafford C. Schulman B. A. (2021). Data-Independent Acquisition Method for Ubiquitinome Analysis Reveals Regulation of Circadian Biology. Nat. Commun. 12, 254. 10.1038/s41467-020-20509-1 33431886 PMC7801436

[B18] Hauser A. S. Attwood M. M. Rask-Andersen M. Schiöth H. B. Gloriam D. E. (2017). Trends in GPCR Drug Discovery: New Agents, Targets and Indications. Nat. Rev. Drug Discov. 16, 829–842. 10.1038/nrd.2017.178 29075003 PMC6882681

[B19] He Q.-T. Xiao P. Huang S.-M. Jia Y.-L. Zhu Z.-L. Lin J.-Y. (2021). Structural Studies of Phosphorylation-Dependent Interactions between the V2R Receptor and Arrestin-2. Nat. Commun. 12, 2396. 10.1038/s41467-021-22731-x 33888704 PMC8062632

[B20] Huang Y. Todd N. Thathiah A. (2017). The Role of GPCRs in Neurodegenerative Diseases: Avenues for Therapeutic Intervention. Curr. Opin. Pharmacol. 32, 96–110. 10.1016/j.coph.2017.02.001 28288370

[B21] Humphrey S. J. Azimifar S. B. Mann M. (2015). High-Throughput Phosphoproteomics Reveals *In Vivo* Insulin Signaling Dynamics. Nat. Biotechnol. 33, 990–995. 10.1038/nbt.3327 26280412

[B22] Imai Y. Soda M. Inoue H. Hattori N. Mizuno Y. Takahashi R. (2001). An Unfolded Putative Transmembrane Polypeptide, Which Can lead to Endoplasmic Reticulum Stress, Is a Substrate of Parkin. Cell 105, 891–902. 10.1016/s0092-8674(01)00407-x 11439185

[B23] Irwin N. Whitaker G. M. Lynn F. C. Mcintosh C. H. S. Accili E. A. (2012). Regulation of GIP and GLP1 Receptor Cell Surface Expression by N-Glycosylation and Receptor Heteromerization. PLoS ONE 7, e32675. 10.1371/journal.pone.0032675 22412906 PMC3296735

[B24] Kallemeijn W. W. Lanyon-Hogg T. Panyain N. Goya Grocin A. Ciepla P. Morales-Sanfrutos J. (2021). Proteome-wide Analysis of Protein Lipidation Using Chemical Probes: In-Gel Fluorescence Visualization, Identification and Quantification of N-Myristoylation, N- and S-Acylation, O-Cholesterylation, S-Farnesylation and S-Geranylgeranylation. Nat. Protoc. 16, 5083–5122. 10.1038/s41596-021-00601-6 34707257

[B25] Kennedy J. E. Marchese A. (2015). Regulation of GPCR Trafficking by Ubiquitin. Prog. Mol. Biol. Transl Sci. 132, 15–38. 10.1016/bs.pmbts.2015.02.005 26055053 PMC4699176

[B26] Kitata R. B. Choong W.-K. Tsai C.-F. Lin P.-Y. Chen B.-S. Chang Y.-C. (2021). A Data-independent Acquisition-Based Global Phosphoproteomics System Enables Deep Profiling. Nat. Commun. 12, 2539. 10.1038/s41467-021-22759-z 33953186 PMC8099862

[B27] Kooistra A. J. Mordalski S. Pándy-Szekeres G. Esguerra M. Mamyrbekov A. Munk C. (2021). GPCRdb in 2021: Integrating GPCR Sequence, Structure and Function. Nucleic Acids Res. 49, D335–D343. 10.1093/nar/gkaa1080 33270898 PMC7778909

[B28] Krishna R. G. Wold F. (1993). Post-Translational Modification of Proteins. Adv. Enzymol. Relat. Areas Mol. Biol. 67, 265–298. 10.1002/9780470123133.ch3 8322616

[B29] Lackman J. J. Goth C. K. Halim A. Vakhrushev S. Y. Clausen H. Petäjä-Repo U. E. (2018). Site-Specific O-Glycosylation of N-Terminal Serine Residues by Polypeptide GalNAc-Transferase 2 Modulates Human δ-Opioid Receptor Turnover at the Plasma Membrane. Cell Signal. 42, 184–193. 10.1016/j.cellsig.2017.10.016 29097258

[B30] Lagerström M. C. Schiöth H. B. (2008). Structural Diversity of G Protein-Coupled Receptors and Significance for Drug Discovery. Nat. Rev. Drug Discov. 7, 339–357. 10.1038/nrd2518 18382464

[B31] Lawrence R. T. Searle B. C. Llovet A. Villén J. (2016). Plug-and-Play Analysis of the Human Phosphoproteome by Targeted High-Resolution Mass Spectrometry. Nat. Methods 13, 431–434. 10.1038/nmeth.3811 27018578 PMC5915315

[B32] Lee S. Park S. Lee H. Han S. Song J. M. Han D. (2019). Nedd4 E3 Ligase and Beta-Arrestins Regulate Ubiquitination, Trafficking, and Stability of the mGlu7 Receptor. Elife 8, e44502. 10.7554/eLife.44502 31373553 PMC6690720

[B33] Lefkowitz R. J. (1998). G Protein-Coupled Receptors. III. New Roles for Receptor Kinases and Beta-Arrestins in Receptor Signaling and Desensitization. J. Biol. Chem. 273, 18677–18680. 10.1074/jbc.273.30.18677 9668034

[B34] Liu J. J. Sharma K. Zangrandi L. Chen C. Humphrey S. J. Chiu Y. T. (2018). *In Vivo* Brain GPCR Signaling Elucidated by Phosphoproteomics. Science 360 (6395), eaao4927. 10.1126/science.aao4927 29930108 PMC6527112

[B35] Liu M.-Q. Zeng W.-F. Fang P. Cao W.-Q. Liu C. Yan G.-Q. (2017). pGlyco 2.0 Enables Precision N-Glycoproteomics with Comprehensive Quality Control and One-Step Mass Spectrometry for Intact Glycopeptide Identification. Nat. Commun. 8, 438. 10.1038/s41467-017-00535-2 28874712 PMC5585273

[B36] Lu H. Fang C. (2020). Methodology for Detecting Protein Palmitoylation. Adv. Exp. Med. Biol. 1248, 425–430. 10.1007/978-981-15-3266-5_17 32185720

[B37] Marchese A. Benovic J. L. (2001). Agonist-Promoted Ubiquitination of the G Protein-Coupled Receptor CXCR4 Mediates Lysosomal Sorting. J. Biol. Chem. 276, 45509–45512. 10.1074/jbc.C100527200 11641392

[B38] Moulédous L. Froment C. Burlet-Schiltz O. Schulz S. Mollereau C. (2015). Phosphoproteomic Analysis of the Mouse Brain Mu-Opioid (MOP) Receptor. FEBS Lett. 589, 2401–2408. 10.1016/j.febslet.2015.07.025 26226422

[B39] Nguyen A. H. Thomsen A. R. B. Cahill T. J. 3rd Huang R. Huang L.-Y. Marcink T. (2019). Structure of an Endosomal Signaling GPCR-G Protein-β-Arrestin Megacomplex. Nat. Struct. Mol. Biol. 26, 1123–1131. 10.1038/s41594-019-0330-y 31740855 PMC7108872

[B40] Nobles K. N. Xiao K. Ahn S. Shukla A. K. Lam C. M. Rajagopal S. (2011). Distinct Phosphorylation Sites on the Beta(2)-Adrenergic Receptor Establish a Barcode that Encodes Differential Functions of Beta-Arrestin. Sci. Signal. 4, ra51. 10.1126/scisignal.2001707 21868357 PMC3415961

[B41] Olsen J. V. Mann M. (2013). Status of Large-Scale Analysis of Post-Translational Modifications by Mass Spectrometry. Mol. Cel Proteomics 12, 3444–3452. 10.1074/mcp.O113.034181 PMC3861698 24187339

[B42] Park D. h. Park S. Song J. m. Kang M. Lee S. Horak M. (2020). N‐Linked Glycosylation of the mGlu7 Receptor Regulates the Forward Trafficking and Transsynaptic Interaction with Elfn1. FASEB j. 34, 14977–14996. 10.1096/fj.202001544R 32931036

[B43] Patwardhan A. Cheng N. Trejo J. (2021). Post-Translational Modifications of G Protein-Coupled Receptors Control Cellular Signaling Dynamics in Space and Time. Pharmacol. Rev. 73, 120–151. 10.1124/pharmrev.120.000082 33268549 PMC7736832

[B44] Percher A. Ramakrishnan S. Thinon E. Yuan X. Yount J. S. Hang H. C. (2016). Mass-Tag Labeling Reveals Site-Specific and Endogenous Levels of Protein S-Fatty Acylation. Proc. Natl. Acad. Sci. USA 113, 4302–4307. 10.1073/pnas.1602244113 27044110 PMC4843475

[B45] Prihandoko R. Bradley S. J. Tobin A. B. Butcher A. J. (2015). Determination of GPCR Phosphorylation Status: Establishing a Phosphorylation Barcode. Curr. Protoc. Pharmacol. 69, 2.13.1–2.13.26. 10.1002/0471141755.ph0213s69 26344213

[B46] Qanbar R. Bouvier M. (2003). Role of Palmitoylation/Depalmitoylation Reactions in G-Protein-Coupled Receptor Function. Pharmacol. Ther. 97, 1–33. 10.1016/s0163-7258(02)00300-5 12493533

[B47] Salom D. Lodowski D. T. Stenkamp R. E. Trong I. L. Golczak M. Jastrzebska B. (2006). Crystal Structure of a Photoactivated Deprotonated Intermediate of Rhodopsin. Proc. Natl. Acad. Sci. 103, 16123–16128. 10.1073/pnas.0608022103 17060607 PMC1637547

[B48] Schjoldager K. T. Narimatsu Y. Joshi H. J. Clausen H. (2020). Global View of Human Protein Glycosylation Pathways and Functions. Nat. Rev. Mol. Cel Biol 21, 729–749. 10.1038/s41580-020-00294-x 33087899

[B49] Shen J. Jia L. Dang L. Su Y. Zhang J. Xu Y. (2021). StrucGP: De Novo Structural Sequencing of Site-Specific N-Glycan on Glycoproteins Using a Modularization Strategy. Nat. Methods 18, 921–929. 10.1038/s41592-021-01209-0 34341581

[B50] Steentoft C. Vakhrushev S. Y. Joshi H. J. Kong Y. Vester-Christensen M. B. Schjoldager K. T.-B. G. (2013). Precision Mapping of the Human O-GalNAc Glycoproteome through SimpleCell Technology. Embo J. 32, 1478–1488. 10.1038/emboj.2013.79 23584533 PMC3655468

[B51] Steentoft C. Vakhrushev S. Y. Vester-Christensen M. B. Schjoldager K. T.-B. G. Kong Y. Bennett E. P. (2011). Mining the O-Glycoproteome Using Zinc-Finger Nuclease-Glycoengineered SimpleCell Lines. Nat. Methods 8, 977–982. 10.1038/nmeth.1731 21983924

[B52] Thomsen A. R. B. Plouffe B. Cahill T. J. 3rd Shukla A. K. Tarrasch J. T. Dosey A. M. (2016). GPCR-G Protein-β-Arrestin Super-Complex Mediates Sustained G Protein Signaling. Cell 166, 907–919. 10.1016/j.cell.2016.07.004 27499021 PMC5418658

[B53] Toghi Eshghi S. Shah P. Yang W. Li X. Zhang H. (2015). GPQuest: A Spectral Library Matching Algorithm for Site-Specific Assignment of Tandem Mass Spectra to Intact N-Glycopeptides. Anal. Chem. 87, 5181–5188. 10.1021/acs.analchem.5b00024 25945896 PMC4721644

[B54] Tsai C.-F. Smith J. S. Krajewski K. Zhao R. Moghieb A. M. Nicora C. D. (2019). Tandem Mass Tag Labeling Facilitates Reversed-phase Liquid Chromatography-Mass Spectrometry Analysis of Hydrophilic Phosphopeptides. Anal. Chem. 91, 11606–11613. 10.1021/acs.analchem.9b01814 31418558 PMC7197904

[B55] Wang T. Nakagawa S. Miyake T. Setsu G. Kunisue S. Goto K. (2020). Identification and Functional Characterisation of N-Linked Glycosylation of the Orphan G Protein-Coupled Receptor Gpr176. Scientific Rep. 10, 4429. 10.1038/s41598-020-61370-y PMC7064540 32157140

[B56] Wilkinson H. Saldova R. (2020). Current Methods for the Characterization of O-Glycans. J. Proteome Res. 19, 3890–3905. 10.1021/acs.jproteome.0c00435 32893643

[B57] Xiao K. Shenoy S. K. (2011). β2-Adrenergic Receptor Lysosomal Trafficking Is Regulated by Ubiquitination of Lysyl Residues in Two Distinct Receptor Domains. J. Biol. Chem. 286, 12785–12795. 10.1074/jbc.M110.203091 21330366 PMC3069478

[B58] Xiao X. Tang J.-J. Peng C. Wang Y. Fu L. Qiu Z.-P. (2017). Cholesterol Modification of Smoothened Is Required for Hedgehog Signaling. Mol. Cel 66, 154–162. 10.1016/j.molcel.2017.02.015 28344083

[B59] Yang W. Ao M. Hu Y. Li Q. K. Zhang H. (2018). Mapping the O-Glycoproteome Using Site-specific Extraction of O-Linked Glycopeptides (EXoO). Mol. Syst. Biol. 14, e8486. 10.15252/msb.20188486 30459171 PMC6243375

[B60] Yang W. Di Vizio D. Kirchner M. Steen H. Freeman M. R. (2010). Proteome Scale Characterization of Human S-Acylated Proteins in Lipid Raft-Enriched and Non-Raft Membranes. Mol. Cel Proteomics 9, 54–70. 10.1074/mcp.M800448-MCP200 PMC2808267 19801377

[B61] Zeng W.-F. Cao W.-Q. Liu M.-Q. He S.-M. Yang P.-Y. (2021). Precise, Fast and Comprehensive Analysis of Intact Glycopeptides and Modified Glycans with pGlyco3. Nat. Methods 18, 1515–1523. 10.1038/s41592-021-01306-0 34824474 PMC8648562

[B62] Zhang Q. Xiao K. Liu H. Song L. Mcgarvey J. C. Sneddon W. B. (2018). Site-Specific Polyubiquitination Differentially Regulates Parathyroid Hormone Receptor-Initiated MAPK Signaling and Cell Proliferation. J. Biol. Chem. 293, 5556–5571. 10.1074/jbc.RA118.001737 29444827 PMC5900769

[B63] Zhou J. Wild C. (2019). GPCR Drug Discovery: Emerging Targets, Novel Approaches and Future Trends. Curr. Top. Med. Chem. 19, 1363–1364. 10.2174/156802661916190828093500 31513505 PMC6905493

[B64] Zhou X. E. He Y. De Waal P. W. Gao X. Kang Y. Van Eps N. (2017). Identification of Phosphorylation Codes for Arrestin Recruitment by G Protein-Coupled Receptors. Cell 170, 457–469. 10.1016/j.cell.2017.07.002 28753425 PMC5567868

[B65] Zielinska D. F. Gnad F. Wiśniewski J. R. Mann M. (2010). Precision Mapping of an *In Vivo* N-Glycoproteome Reveals Rigid Topological and Sequence Constraints. Cell 141, 897–907. 10.1016/j.cell.2010.04.012 20510933

[B66] Zuckerman D. M. Hicks S. W. Charron G. Hang H. C. Machamer C. E. (2011). Differential Regulation of Two Palmitoylation Sites in the Cytoplasmic Tail of the β1-Adrenergic Receptor. J. Biol. Chem. 286, 19014–19023. 10.1074/jbc.M110.189977 21464135 PMC3099716

